# *RAS* and *TP53* can predict survival in adults with T‐cell lymphoblastic leukemia treated with hyper‐CVAD

**DOI:** 10.1002/cam4.2757

**Published:** 2019-12-05

**Authors:** Ali Sakhdari, Beenu Thakral, Sanam Loghavi, Rashmi Kanagal‐Shamanna, C. Cameron Yin, Zhuang Zuo, Mark J. Routbort, Rajyalakshmi Luthra, L. Jeffrey Medeiros, Sa A. Wang, Keyur P. Patel, Chi Young Ok

**Affiliations:** ^1^ Department of Hematopathology The University of Texas MD Anderson Cancer Center Houston TX USA

**Keywords:** *RAS*, risk stratification, T‐cell acute lymphoblastic leukemia, *TP53*

## Abstract

Adult T‐cell acute lymphoblastic leukemia (T‐ALL) is a heterogeneous group of acute leukemias that account for about one third of all cases of Philadelphia chromosome (Ph)‐negative ALL. Recently, a molecular classifier using the mutational status of *NOTCH1*, *FBXW7*, *RAS*, and *PTEN* (NFRP) has been shown to distinguish low‐ vs high‐risk groups in adult T‐ALL patients treated using the Berlin‐Frankfurt‐Münster ALL protocol. However, it is unknown if this molecular classifier can stratify adult T‐ALL patients treated with hyper‐CVAD ± nelarabine. We identified a relatively small cohort of 27 adults with T‐ALL who were uniformly treated with hyper‐CVAD ± nelarabine with available mutational analysis at time of diagnosis. The most commonly mutated genes in this group were *NOTCH1* (52%), *NRAS* (22%), *DNMT3A* (19%), *KRAS* (15%), and *TP53* (7%). The NFRP molecular classifier failed to stratify overall survival (OS; *P* = .84) and relapse‐free survival (RFS; *P* = .18) in this cohort. We developed a new stratification model combining *K/NRAS* and *TP53* mutations as high‐risk factors and showed that mutations in these genes predicted poorer OS (*P* = .03) and RFS (*P* = .04). While the current study is limited by cohort size, these data suggest that the NFRP molecular classifier might not be applicable to adult T‐ALL patients treated with hyper‐CVAD ± nelarabine. *RAS/TP53* mutation status, however, was useful in risk stratification in adults with T‐ALL.

## Graphical abstract

The NFRP (NOTCH1/FBXW7/RAS/PTEN) risk stratification is not optimal for T‐ALL patients treated with the hyper‐CVAD regimen. The new MDACC (TP53/RAS) risk stratification can identify high‐ and low‐risk groups in hyper‐CVAD‐treated patients.

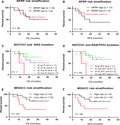

## 1 INTRODUCTION

T‐cell acute lymphoblastic leukemia (ALL)/lymphoma is an uncommon disease in adults and more aggressive than the more common pediatric counterpart.^1^ T‐ALL in adults, however, is potentially curable with 50% of 5‐year survival rate.^2^ Chromosomal translocations occur in a subset T‐ALL cases; these translocations often involve the T‐cell receptor gene loci or *KMT2A* with variable partner genes, including *TAL1*, *TAL2*, *TLX1*, *TLX3*, *HOXA*, *LMO1*, *LMO2*, and *NKX2*.^3^, ^4^, ^5^, ^6^, ^7^ Several genes involving various cellular signaling pathways are also recurrently mutated in T‐ALL. Examples of these mutations are *PTEN* mutation/deletion in PI3K‐AKT pathway and *N/KRAS* mutations in MAPK‐ERK signaling pathway.^8^, ^9^, ^10^ Activation of NOTCH1 pathway is also a hallmark of both pediatric and adult T‐ALL implicating a favorable outcome.^11^, ^12^, ^13^ In most instances NOTCH1 activation results from activating mutations in *NOTCH1* but in fewer cases loss‐of‐function mutations in *FBXW7*, an inhibitor of *NOTCH1*, lead to constitutive NOTCH1 overexpression.^14^, ^15^ Various combinations of common gene alterations in T‐ALL have been associated with different responses to therapy and different clinical outcomes.^8^, ^16^, ^17^, ^18^, ^19^

The overall outcome in adult T‐ALL has improved over the past several decades, largely due to better risk stratification and intensified chemotherapeutic regimens.^20^, ^21^, ^22^ Major prognostically important clinical factors in T‐ALL patient are age at diagnosis, peripheral blood (PB) count (tumor burden), maturational stage of neoplastic cells and CNS involvement.^2^, ^23^ Status of minimal residual disease (MRD) is considered the single most influential factor in predicting long‐term survival after induction therapy.^24^, ^25^, ^26^, ^27^, ^28^

Several large scale studies have shown clinically relevant genetic changes in both pediatric and adult T‐ALL.^9^, ^11^, ^18^, ^19^, ^29^, ^30^, ^31^ Trinquand et al suggested that a *NOTCH1/FBXW7/RAS/PTEN*‐based classifier predicts relapse‐free survival (RFS) and overall survival (OS) in adults with T‐ALL.^29^ The utility of this approach was further confirmed in children with T‐ALL.^32^, ^33^ In this model, T‐ALL with mutations in *NOTCH1/FBXW7* (*N/F*) without any changes in either *(K/N)RAS* or *PTEN* (*R/P*) is considered a genetically low‐risk group, whereas all other combinations of these gene mutations were considered genetically high‐risk.^29^ The induction chemotherapeutic regimen used in previous studies for this oncogenetic classifier consisted of vincristine, daunorubicin, L‐asparaginase, and cyclophosphamide (Berlin‐Frankfurt‐Münster [BFM] protocol).^29^, ^33^ The standard treatment regimen for adults with T‐ALL at our institution, however, is *h*yper‐fractionated *c*yclophosphamide, *v*incristine, *d*oxorubicin and *d*examethasone (hyper‐CVAD) with or without nelarabine (https://clinicaltrials.gov/ct2/show/NCT00501826).^34^ The reproducibility of (*N/F/R/P*) risk stratification model in adult T‐ALL patients treated with hyper‐CVAD ± nelarabine has not been evaluated. In this study, we assessed the applicability of this model in our cohort of adults with T‐ALL treated with hyper‐CVAD ± nelarabine.

## 2 MATERIALS AND METHODS

### 2.1 Patients

We searched the electronic medical record to identify adult patients with T‐ALL in the bone marrow (BM) between 2012 and 2018. Inclusion criteria included: (a) patients ≥18 years; (b) those who were treatment‐naïve at time of first presentation to our institution; (c) patients treated with hyper‐CVAD ± nelarabine; and (d) next‐generation sequencing (NGS)‐based mutation analysis was performed. Exclusion criteria included: (a) blast crisis of chronic myeloid leukemia with T‐lymphoblasts; (b) mixed phenotype acute leukemia; (c) patients with nodal or extranodal involvement by T‐lymphoblastic lymphoma with minimal (≤5% blasts) BM involvement.

The clinicopathologic, cytogenetic and mutational data on patients in the study were collected by reviewing patients’ electronic medical records. Complete remission (CR) or CR with incomplete hematologic recovery (CRi) were assessed according to the latest national comprehensive cancer network clinical practice guidelines.^35^ Qualitative polymerase chain reaction‐based methods were performed using genomic DNA (gDNA) extracted from BM aspirate specimens to assess for rearrangements of *TRG* and *TRB*.^36^ Measurable MRD was analyzed by multiparameter flow cytometry (MFC) analyses (assay has been validated to a sensitivity of 0.1%‐0.01%). This study was approved by the institutional Review Board at The University of Texas MD Anderson Cancer Center and performed in accord with the Declaration of Helsinki.

### 2.2 NGS analysis

Next‐generation sequencing‐based mutation analysis was performed using previously described 28‐gene or 81‐gene panels (complete list of the genes in Table S1).^37^ Briefly, sequencing libraries were prepared from 250 ng of gDNA using HaloPlex Target Enrichment Kit (Agilent Technologies) and sequencing libraries were subject to a MiSeq sequencer (Illumina). NGS data analysis was performed using SureCall (Haloplex). The Integrative Genomics Viewer (IGV; Broad Institute) was used to visualize read alignment and confirm variant calls.^38^ A custom‐developed, in‐house software package (OncoSeek) was used to annotate sequence variants and to interface the data with the IGV. Nomenclature of genetic variants was designated following the Human Genome Variation Society recommendations.^39^ The limit of detection of the NGS assays was 1%.

### 2.3 Statistical analysis

Overall survival was defined from the time of diagnosis to death from any cause. RFS and time to relapse were defined as the time from diagnosis or remission (CR/CRi) to first outcome event (induction failure, death during remission, or relapse), respectively. Patients who underwent stem cell transplant were censored. Statistical analysis was performed using GraphPad Prism 7 (GraphPad Software, Inc) and IBM^®^ SPSS Statistics 24 (IBM, Inc). Fisher's exact test and Mann‐Whitney *U* test were used to assess categorical and continuous variables, respectively. Survival probability was determined using the Kaplan‐Meier method, with difference compared by the log‐rank test. A Cox proportional‐hazards model was used for univariate and multivariate analysis. A *P*‐value (two‐sided) under .05 was considered statistically significant.

## 3 RESULTS

### 3.1 Patient characteristics

The study cohort includes 27 patients, 23 men and 4 women, with the median age at diagnosis of 37 years (range: 18‐75 years) (Table 1). The median hemoglobin level was 10.3 g/dL (range: 5.8‐16.9 g/dL); leukocyte count 14.5 × 10^9^/L (range: 1‐137 × 10^9^/L), and platelet count 123 × 10^9^/L (range: 13‐327 × 10^9^/L). The median blast count was 80% (range: 6%‐96%) and 61% (range: 0%‐100%) in BM and PB, respectively. Immunophenotype included early T‐cell precursor (n = 9), double negative (n = 8), double positive (n = 4), and single positive (n = 6).

**Table 1:** Clinical and laboratory characteristics of patient cohort

Feature	T‐cell acute lymphoblastic leukemia			
	Total (n = 27)	Low risk (n = 18) [w/o. *RAS* or *TP53* mut]	High risk (n = 9) [w. *RAS* or *TP53* mut]	*P*‐value
Gender				
Male	23	18	7	.57
Female	4	2	2	
Median age (y) (range)	37 (18‐75)	42 (20‐70)	26 (18‐75)	.56
White blood cell count (×10^9^/L) (range)	14.5 (1‐137)	28.5 (2‐108)	9.3 (1‐137)	.77
Platelet (×10^3^/µL) (range)	123 (13‐327)	177.5 (13‐327)	53 (13‐203)	.06
Hg (g/dL) (range)	10.3 (5.8‐16.9)	10.4 (5.8‐16.9)	10.2 (7.6‐15.4)	.80
Blast % (range)				
Bone marrow	80 (6‐96)	75.5 (6‐94)	82 (45‐96)	.19
Peripheral blood	61 (0‐100)	65.5 (0‐100)	32 (0‐90)	.63
Cytogenetic (n = 25)				
Normal	12	8	4	.99
Simple	4	3	1	
Complex	9	6	3	
TR gene subsets				
Gamma only	5	4	1	.99
Beta	14	8	6	
Germline	6	4	2	
3‐y overall survival	50%	72%	36%	.02

Conventional cytogenetic analysis was available in 25 patients. These included 12 patients with normal karyotype, 4 with a simple abnormality (<3 abnormalities), and 9 with a complex karyotype (≥3 abnormalities). Well‐known translocations involving T‐cell receptor gene loci, t(10;11)(p13;q14), or t(11;19)(q23;p13) were not present. Monoclonal T‐cell receptor gene rearrangements (*TRG* and/or *TRB*) were detected in 19 (76%) patients. All patients were treated with the standard chemotherapy regimen of hyper‐CVAD (n = 6) or hyper‐CVAD + nelarabine (n = 21).

### 3.2 High rate of complete remission (CR/CRi) after hyper‐CVAD ± nelarabine regimen

Twenty‐six (96%) patients achieved CR/CRi after the first or second course of induction chemotherapy. Eight of 26 (31%) patients relapsed at a median interval of 9.3 months (range: 3.2‐18.2 months) after remission. With a median follow‐up of 22.6 months (range: 3.8‐49.7 months), 15 (65%) patients were alive and the 3‐year OS rate was 50%. The median OS was 32.6 months.

### 3.3 Commonly mutated genes in T‐ALL

Twenty‐six (96%) patients had mutations in at least one of the tested gene. Fourteen (52%) patients showed a total of 19 *NOTCH1* mutations. Recurrent hotspot mutations were not seen in *NOTCH1*. The median mutant allelic frequency (MAF) was 29% (range: 2.3%‐53.5%) indicating a heterozygous change in most cases. Six patients had *NOTCH1* mutation with a MAF < 10%. Three of these patients had other major mutant clones in *NOTCH* 1 (patients #2 and 4) and *TP53* (patient #7), respectively. *NOTCH1* mutation was the only mutation in the remaining three patients (patients #8, 10, and 12) who had 81%, 32%, and 24% blasts in bone marrow, respectively.

Other recurrently mutated genes in this cohort were *NRAS* (n = 6), *DNMT3A* (n = 5), *KRAS* (n = 4) and *TP53* (n = 2) (Table 2). The median MAF of the *NRAS* mutations was 37.8% (range: 3.6%‐48.2%). Two patients had *NRAS* mutation with a MAF < 10%; both had major mutant clones in *NOTCH1* (patients #2 and 4). Five of 6 patients with *NRAS* mutation also had a *NOTCH1* mutation. The median MAF of *DNMT3A* mutation was 41.5% (range: 2.6%‐49.8%). None of the 5 patients with *DNMT3A* mutation had a *NOTCH1* mutation. *KRAS* mutations were mostly subclonal (median MAF: 5.5%) and 2 of 4 *KRAS*‐mutated patients with MAF < 10% had mutations in other genes. In contrast, *TP53* mutations were major clones (MAF: 93.5% and 25.4%). The two patients with *TP53* mutation also had *NOTCH1* mutation. Mutations in *FBXW7* and *PTEN* were not detected in the study cohort.

**Table 2:** Most commonly mutated genes in our patient cohort at the time of diagnosis. The mutant allele frequency (MAF) of mutated genes is indicated inside the corresponding box

Gene	Pt.ID																										
	1	2	3	4	5	6	7	8	9	10	11	12	13	14	15	16	17	18	19	20	21	22	23	24	25	26	27
*NOTCH1*	32.1	38.1/5.3	53.5	35.9/2.3	38.3	48.3/42.4	4.4	9.1	13.3	6.6	46/29	5.5	23.4	23.3													
*NRAS*	18.2	4.5	48.2	37.8/3.6	46.3										39.3												
*KRAS*	9.4	40.9													1.7	1.2											
*DNMT3A*															46/41.7		19.2	2.6	41.2	49.8							
*TP53*						93.8	25.4																				

### 3.4 N/F/R/P binary risk model did not stratify T‐ALL patients treated with hyper‐CVAD‐based regimen

**Figure 1:**
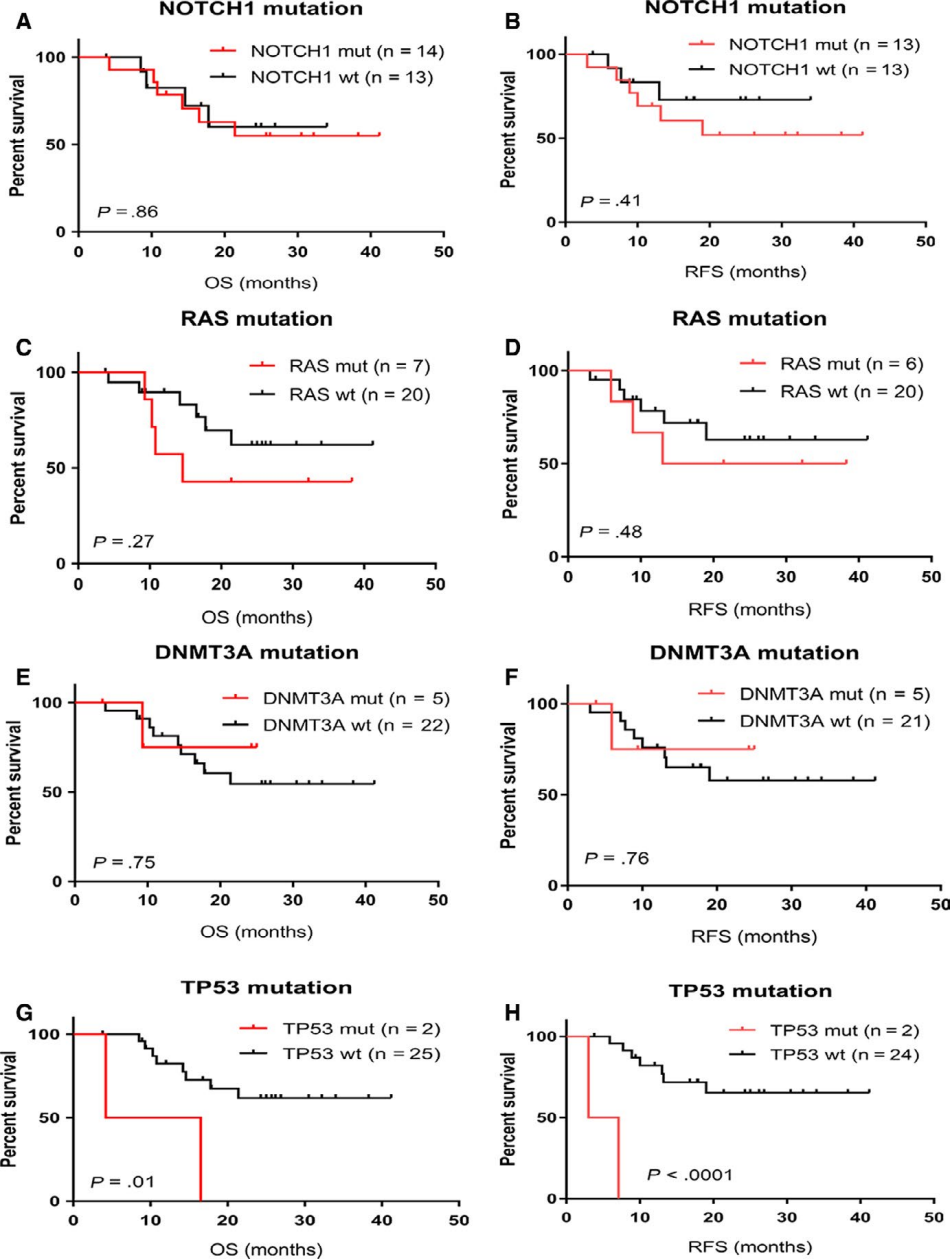
Probability of OS and RFS in our patient cohort based on multiple single gene mutations at the time of diagnosis. A and B, OS and RFS regarding *NOTCH1* mutation. C and D, OS and RFS regarding *K/RAS* mutation. E and F, OS and RFS regarding *DNMT3A *mutation. G and H, OS and RFS regarding regarding *TP53 *mutation. Only a mutated *TP53* showed a significant effect on both OS and RFS. mut, mutated; OS, overall survival; RFS, relapse free survival; wt, wildtype

**Figure 2:**
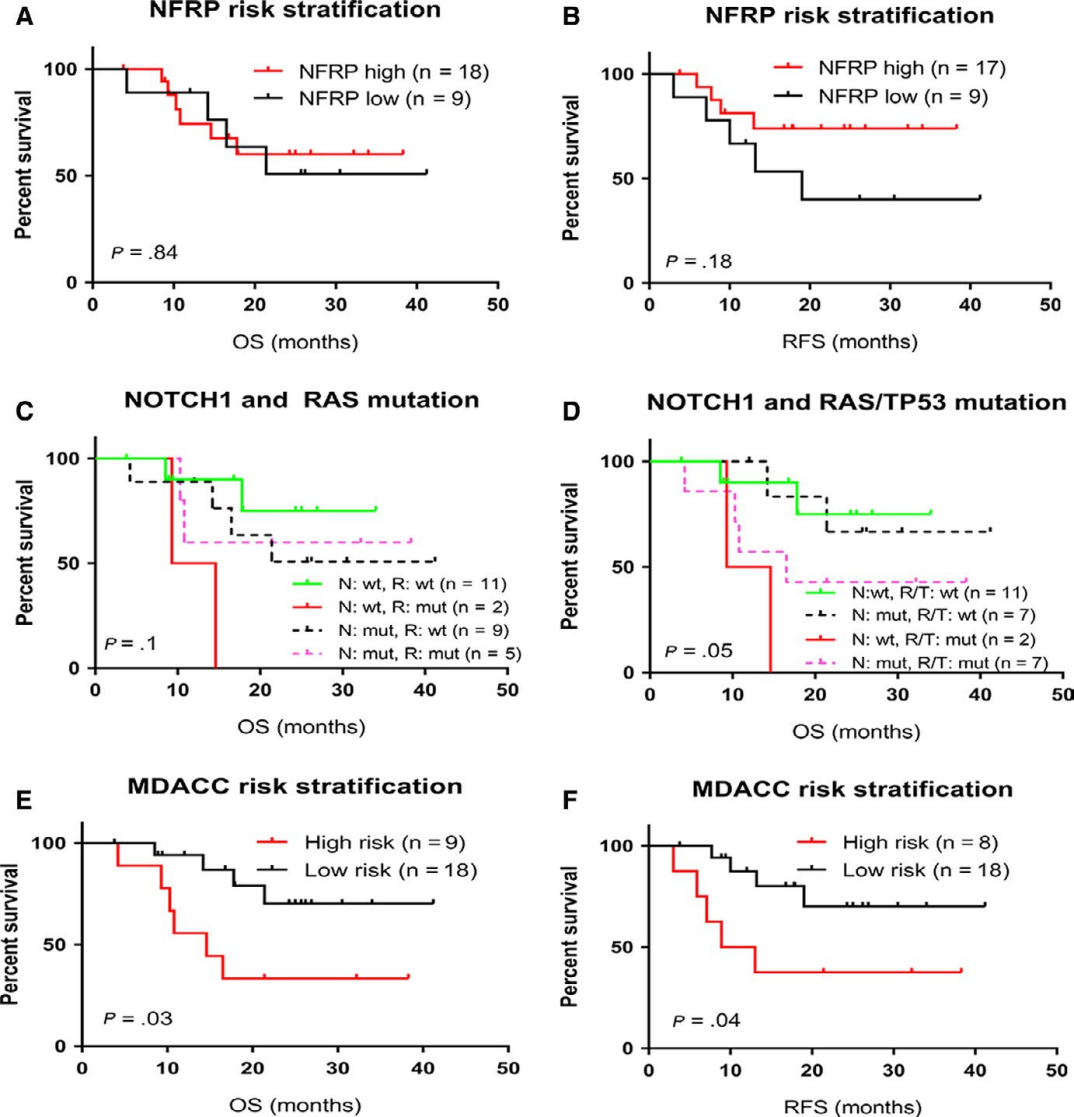
Probability of OS and RFS in our patient cohort based on different combinations of gene mutations at the time of diagnosis. A and B, Low‐risk and high‐risk groups were determined according to the NFRP model (low risk: *NOTCH1/FBXW7* mutated and *RAS/PTEN* wildtype and; high risk: *RAS* or *PTEN* mutated). C, Different combinations of *NOTCH1* and *(N/K)RAS* mutations. D, Different combinations of *NOTCH1* and combined (*RAS* or *TP53*) gene mutations. E and F, Low‐risk and high‐risk groups were determined according to the MDACC model (low risk: *RAS* or *TP53* wild‐type; high risk: *RAS* and/or *TP53* mutation). MDACC, MD Anderson Cancer Center; mut, mutated; NFRP, *NOTCH*, *FBXW7*, *RAS*, *PTEN*; OS, overall survival; R/T, *RAS* or *TP53*; RFS, relapse free survival; wt, wildtype

As a single mutation, no significant differences in outcome were observed in patients with *NOTCH1*, *NRAS*, or *DNMT3A* mutation (Figure 1A‐F). However, patients with *TP53* mutation had a poor outcome (Figure 1G,H). Meanwhile, the N/F/R/P binary risk model suggested by Trinquand et al^29^ failed to adequately stratify the patients in this cohort (Figure 2A,B). We further analyzed survival outcome of 4 groups in this cohort based on the mutational status of *NOTCH1* and *RAS*, which did not demonstrate satisfactory risk stratification (Figure 2C). In the patient group with wild‐type *NOTCH1*, the presence of *RAS* mutation predicted a poorer prognosis (*P* = .01). In the group with *NOTCH1* mutation, however, outcome was similar irrespective of *RAS* mutation (*P* = .93). Given the fact that *TP53* mutation was co‐mutated with *NOTCH1*, we re‐classified the group based on *NOTCH1* and *RAS*/*TP53* mutations. The new 4‐group risk model showed improved stratification in outcome (Figure 2D). Since *NOTCH1* mutation did not show much difference in *RAS*/*TP53* wild‐type group and mutated patient groups, we further simplified stratification of patients into 2 groups based on *RAS*/*TP53* mutation irrespective of *NOTCH1* status (low‐risk [n = 18]: *RAS* and *TP53* wild‐type, high‐risk [n = 9]: *RAS* or *TP53* mutated, hereafter will be referred to MDACC risk groups). This new risk model showed significant risk stratification in both OS (*P* = .03) and RFS (*P* = .04) (Figure 2E,F).

### 3.5 End‐of‐induction measurable residual disease (MRD) by flow cytometry did not predict patient outcome

**Figure 3:**
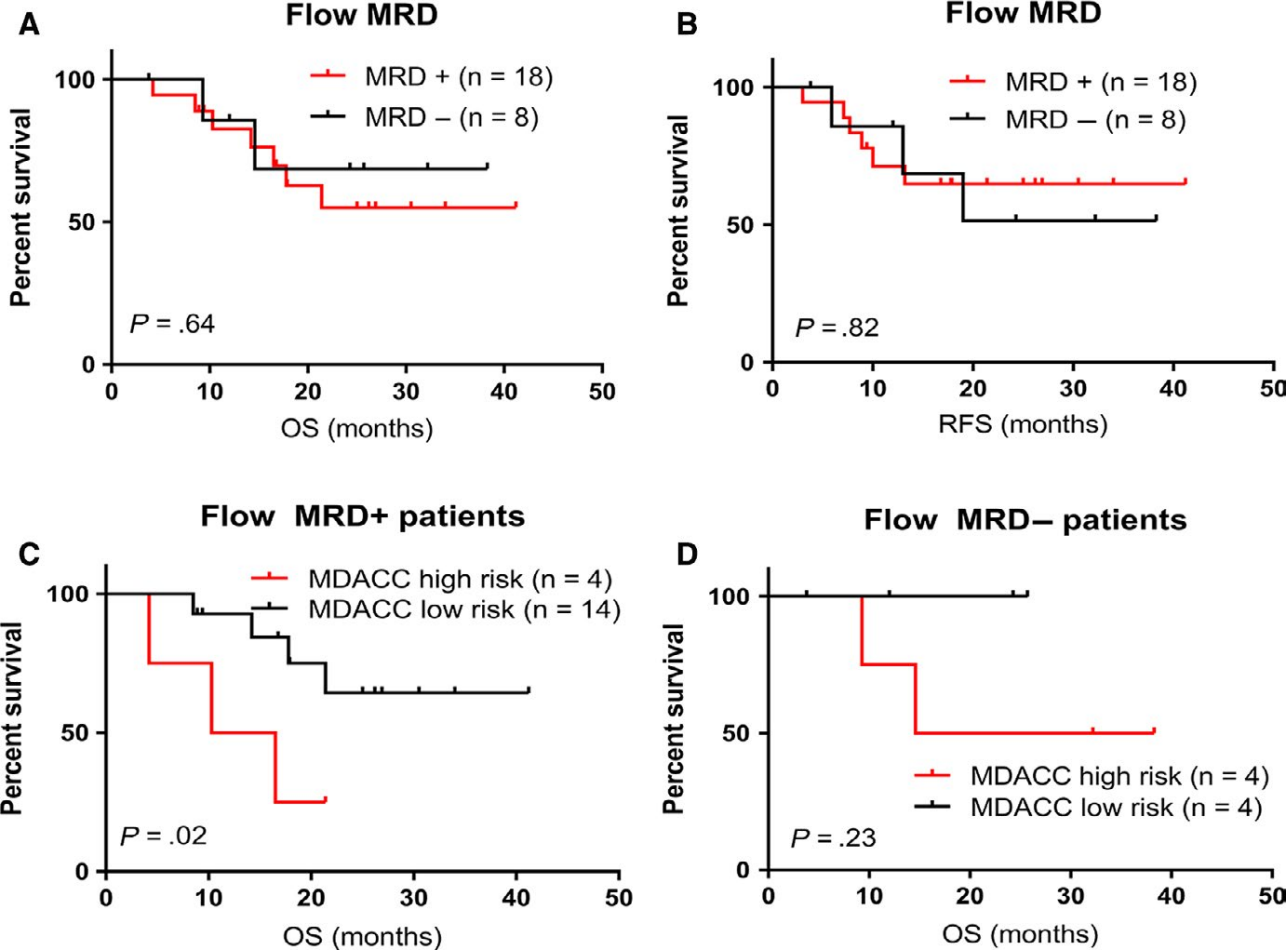
A and B, OS and RFS for patients with T‐ALL based on measurable MRD in all patients. C and D, OS in patients with a positive (C) or a negative (D) MRD at the end of induction chemotherapy stratified based on MDACC risk classifier. MDACC, MD Anderson Cancer Center; MRD, minimal residual disease; OS, overall survival; RFS, relapse free survival; T‐ALL, T‐cell acute lymphoblastic leukemia

The status of MRD was assessed with MFC at the end of first and/or second induction in all but one patient who had refractory disease. Eighteen (69%) and 8 (31%) patients showed a positive and negative MRD at the end of induction chemotherapy. The status of MRD by MFC did not demonstrate significant difference in survival (Figure 3A,B). MDACC risk model further separated two prognostically different groups both in patients with positive MRD (*P* = .02), but not in those with negative MRD (*P* = .23) (Figure 3C,D).

### 3.6 Low white blood cell counts are associated with poor OS in T‐ALL

**Figure 4:**
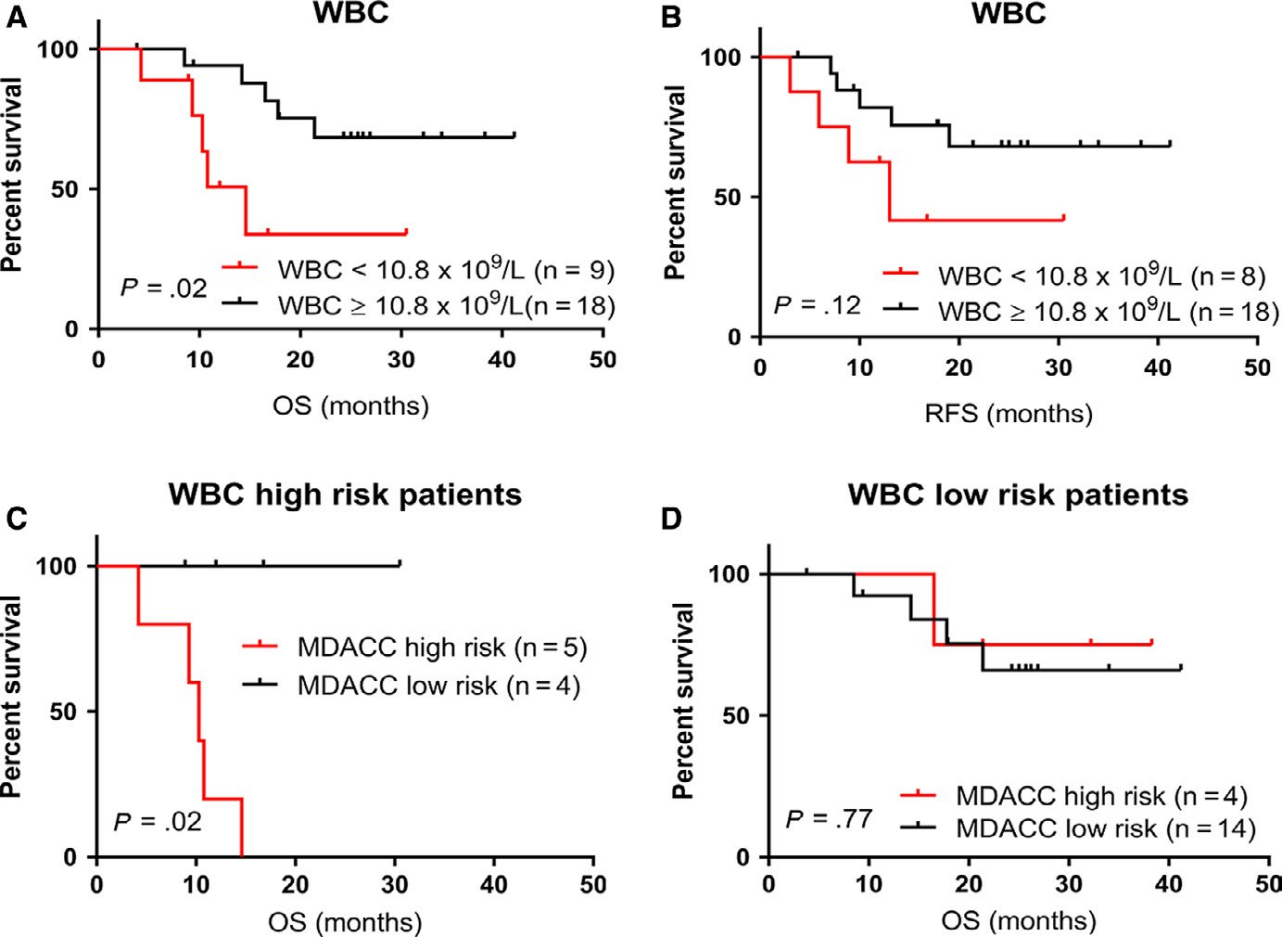
A and B, OS and RFS for patients with T‐ALL based on WBC count at the time of diagnosis in all patients. C and D, OS in patients with a high [≥10.8 × 10^9^/L] (C) or low [<10.8 × 10^9^/L] (D) WBC at the end of induction chemotherapy stratified based on MDACC risk classifier. MDACC, MD Anderson Cancer Center; OS, overall survival; RFS, relapse free survival; T‐ALL, T‐cell acute lymphoblastic leukemia; WBC, white blood cell

Prognostic impact of white blood cell count (WBC) is less firmly established for adult T‐ALL than for the pediatric T‐ALL. High WBC of ≥100 × 10^9^/L, however, is commonly considered a high‐risk factor for both adult and pediatric T‐ALL.^35^ In our cohort the median WBC was 14.5 × 10^9^/L (range: 1‐137 × 10^9^/L) and only two patients (# 6 and 26) had WBC > 100 × 10^9^/L at the time of diagnosis. Due to the skewed distribution to the lower WBC (<100 × 10^9^/L), we performed an ROC curve calculation to select a cutoff of WBC for which the difference in survival is more significant. The WBC of 10.8 × 10^9^/L shows the best discrimination. Based on the new discriminator, patients with WBC of <10.8 × 10^9^/L (n = 9) had worse outcome compared with those with higher WBC (≥10.8 × 10^9^/L) (n = 18) (median OS: 14.6 months and not reached, respectively, *P* = .02; median RFS: 13 months and not reached, respectively, *P* = .12) (Figure 4A,B). Similar to the above subgroup analysis with respect to MRD status, MDACC risk model further separated two prognostically different groups both in patients with WBC of <10.8 × 10^9^/L (*P* = .02), but not in those with WBC of ≥10.8 × 10^9^/L (*P* = .77) (Figure 4C,D).

### 3.7 MDACC risk stratification is an independent factor predicting worse OS in adult T‐ALL patients treated with the hyper‐CVAD

In univariate analysis, both MDACC high risk and lower WBC had increased risk of death. MRD status did not show any statistically significant difference. In multivariate analysis, MDACC risk model remained to have an increased risk of death (hazard ratio; 4.9, 95% confidence interval; 1.213‐19.621, *P* = .026) (Table 3).

**Table 3:** Specific hazard ratios (HR) calculated in univariate and multivariate analysis of three major factors of MDACC risk stratification (MDACC), white blood cells counts (WBC), and measurable residual disease (MRD) historically important in prognosis of T acute lymphoblastic leukemia for overall survival (OS)

Variable	Univariate analysis			Multivariate analysis		
	HR	95% CI	*P*‐value	HR	95% CI	*P*‐value
MDACC	3.663	1.027‐13.070	.045	4.878	1.213‐19.621	.026
WBC	4.13	1.121‐15.219	.033	3.992	1.002‐15.903	.050
MRD	1.62	0.343‐7.637	.542	3.068	0.596‐15.798	.180

## 4 DISCUSSION

In this study, we have examined a relatively small cohort of uniformly treated adult T‐ALL patients for whom a systematic mutation analysis for the most relevant genes in T‐ALL were performed at the time of diagnosis and before the initiation of induction therapy. While studying such a homogenous group of patients from a rare entity such as T‐ALL is valuable, it should be clarified that the outcome of the study is considered preliminary due to low number of patients in the cohort.

The mutational profile of adult T‐ALL in our cohort is similar to that reported in the literature.^14^, ^40^ Almost all patients in our cohort had a mutation(s) in at least one gene. *NOTCH1* was the most common gene mutation, in over half of cases, followed by *KRAS/NRAS* and *DNMT3A* mutations in 26%, and 19%, respectively. *NOTCH1* mutations were usually a major clone (MAF ≥ 10%), but subclonal fraction (MAF < 10%) was not uncommon. *NRAS* mutation (22%) was more common than *KRAS* mutation (15%), but co‐mutations in both *NRAS* and *KRAS* were found in 50% and 75% of *NRAS*‐ and *KRAS*‐mutated T‐ALL cases. Unlike other studies, *DNMT3A* mutation was mutually exclusive to *NOTCH1* mutation in this cohort.^41^ *TP53* mutation was rare in this study (2/27, 7%) which is similar to the frequencies identified in previous studies with much larger cohort of adult T‐ALL patients (between 5% and 11%).^42^, ^43^ We did not observe any alterations in the *FBXW7* and *PTEN* genes.

*NOTCH1* mutation has been associated with a favorable outcome in most of earlier studies.^11^, ^12^, ^13^, ^14^ However, in this study we did not observe a favorable outcome for patients with *NOTCH1* mutation (Figure 1A,B). We also analyzed patients with a major *NOTCH1* mutant clone (MAF > 10%), but a favorable outcome was not observed (data now shown). As the presence of mutations in *TP53* 42, 43 or *RAS* 17 at the time of diagnosis of T‐ALL have been reported to be correlated with an unfavorable outcome, and seven of 14 *NOTCH1*‐mutated patients also had mutations of *K/NRAS* or *TP53*, we speculated that this unexpected negative result may be due to presence of the co‐mutation. Nevertheless, exclusion of *TP53*‐ or *K/NRAS*‐mutated cases did not reveal any favorable outcome for *NOTCH1* mutated cases (data not shown). Similarly, *RAS* mutation was not associated with a poorer outcome in our cohort (Figure 1C,D), which showed prognosis in other studies.^17^, ^44^ Furthermore, when we applied the N/F/R/P classifier, we did not observe any prognostic discrimination in our cohort (Figure 2A,B). However, when we divided our patients into 4 groups based on wild‐type or mutated *NOTCH1* and *RAS*, it did not show satisfactory stratification (*P* = .1) (Figure 2C). Focusing on the *NOTCH1* wild‐type subgroup, the presence of a *RAS* mutation showed a poorer prognosis (*P* = .01). However, in the *NOTCH1*‐mutated subgroup, outcome was similar irrespective of *RAS* mutation (*P* = .93). We noticed that *TP53*‐mutated patients in our cohort also had co‐mutation in *NOTCH1*. We hypothesized that *TP53* mutation could have negative effect on survival in *NOTCH1*‐mutated patients, and re‐classified our cohort based on *NOTCH1* and *RAS/TP53* mutation. This approach demonstrated improved risk stratification (*P* = .05) (Figure 2D). In this stratification model, *NOTCH1* mutation did not further stratify patients in *RAS*/*TP53*‐mutated (*P* = .89) and *RAS*/*TP53* wild‐type groups (*P* = .17). Therefore, we further simplified the risk model using only *RAS* and *TP53* mutation (low risk: *RAS* and *TP53* wild type, high risk: *RAS* or *TP53* mutation), which showed improved risk stratification compared to the N/F/R/P model.

The N/F/R/P classifier failed to stratify T‐ALL patients in our cohort. Presumably, the main reason is due to the fact that *NOTCH1* mutation was not associated with favorable outcome in our cohort. Although reasons are unclear, the prognostic effect of *NOTCH1* mutation in T‐ALL might not be significant if patients are treated with non‐BFM protocols. Indeed, lack of favorable outcome for *NOTCH1* mutation in T‐ALL has been reported, particularly in studies treated with regimens other than BFM‐ALL protocols.^45^, ^46^, ^47^, ^48^ Enrichment of ETP (33%) in our cohort could be attributable to the negative impact of *NOTCH1* mutation since it is well‐known for worse clinical outcome.^49^, ^50^ However, to the best of our knowledge, it is unknown if N/F/R/P classifier retains prognostic power after stratified by immunophenotype. Cytogenetic aberrations do not seem to affect the result since our cohort demonstrates similar cytogenetic profile to previous studies.^49^, ^51^ Instead, *RAS* and *TP53* mutation could be the most significant factor for risk stratification.

Measurable residual disease status measured by flow cytometry after induction chemotherapy was not correlated with outcome in our cohort. However, application of our molecular risk model further identified patients with higher risk, showing the utility of our model. We found an inverse association between white blood cell count and outcome in adult T‐ALL. Patients who had WBC of <10.8 × 10^9^/L showed a significantly poorer OS compared to patient with higher WBC. Similar to MRD status, our molecular risk model further discriminated patients with WBC of <10.8 × 10^9^/L. In univariate analysis, both MDACC molecular risk model and WBC count were significant risk factors but the former remains significant in multivariate analysis.

In summary, the N/F/R/P molecular classifier at diagnosis cannot be applied to adult T‐ALL patients treated with hyper‐CVAD with or without nelarabine. Instead, we found that *RAS* and *TP53* mutations (MDACC risk model) showed improved stratification in adult T‐ALL patients. The poor outcome of *TP53* mutated T‐ALL is in contrast to a recent report showed lack of the MDACC risk model was an independent risk factor in multivariate analysis. A larger, independent study is needed to confirm out data.

## CONFLICT OF INTEREST

All authors have nothing to disclose.

## References

[1] Dores GM , Devesa SS , Curtis RE , Linet MS , Morton LM . Acute leukemia incidence and patient survival among children and adults in the United States, 2001–2007. Blood. 2012;119(1):34‐43. 22086414 10.1182/blood-2011-04-347872 PMC3251235

[2] Marks DI , Rowntree C . Management of adults with T‐cell lymphoblastic leukemia. Blood. 2017;129(9):1134‐1142. 28115371 10.1182/blood-2016-07-692608

[3] De Keersmaecker K , Ferrando AA . TLX1‐induced T‐cell acute lymphoblastic leukemia. Clin Cancer Res. 2011;17(20):6381‐6386. 21705452 10.1158/1078-0432.CCR-10-3037

[4] Van Vlierberghe P , Homminga I , Zuurbier L , et al. Cooperative genetic defects in TLX3 rearranged pediatric T‐ALL. Leukemia. 2008;22(4):762‐770. 18185524 10.1038/sj.leu.2405082

[5] Meijerink JP , Cante‐Barrett K , Vroegindeweij E , Pieters R . HOXA‐activated early T‐cell progenitor acute lymphoblastic leukemia: predictor of poor outcome? Haematologica. 2016;101(6):654‐656. 27252509 10.3324/haematol.2016.145391 PMC5013942

[6] Bergeron J , Clappier E , Cauwelier B , et al. HOXA cluster deregulation in T‐ALL associated with both a TCRD‐HOXA and a CALM‐AF10 chromosomal translocation. Leukemia. 2006;20(6):1184‐1187. 16572206 10.1038/sj.leu.2404187

[7] Curtis DJ , McCormack MP . The molecular basis of Lmo2‐induced T‐cell acute lymphoblastic leukemia. Clin Cancer Res. 2010;16(23):5618‐5623. 20861166 10.1158/1078-0432.CCR-10-0440

[8] Palomero T , Sulis ML , Cortina M , et al. Mutational loss of PTEN induces resistance to NOTCH1 inhibition in T‐cell leukemia. Nat Med. 2007;13(10):1203‐1210. 17873882 10.1038/nm1636 PMC2600418

[9] Gutierrez A , Sanda T , Grebliunaite R , et al. High frequency of PTEN, PI3K, and AKT abnormalities in T‐cell acute lymphoblastic leukemia. Blood. 2009;114(3):647‐650. 19458356 10.1182/blood-2009-02-206722 PMC2713461

[10] Zhang JH , Ding L , Holmfeldt L , et al. The genetic basis of early T‐cell precursor acute lymphoblastic leukaemia. Nature. 2012;481(7380):157‐163. 22237106 10.1038/nature10725 PMC3267575

[11] Asnafi V , Buzyn A , Le Noir S , et al. NOTCH1/FBXW7 mutation identifies a large subgroup with favorable outcome in adult T‐cell acute lymphoblastic leukemia (T‐ALL): a Group for Research on Adult Acute Lymphoblastic Leukemia (GRAALL) study. Blood. 2009;113(17):3918‐3924. 19109228 10.1182/blood-2008-10-184069

[12] Baldus CD , Thibaut J , Goekbuget N , et al. Prognostic implications of NOTCH1 and FBXW7 mutations in adult acute T‐lymphoblastic leukemia. Haematologica. 2009;94(10):1383‐1390. 19794083 10.3324/haematol.2008.005272 PMC2754954

[13] Aifantis I , Raetz E , Buonamici S . Molecular pathogenesis of T‐cell leukaemia and lymphoma. Nat Rev Immunol. 2008;8(5):380‐390. 18421304 10.1038/nri2304

[14] Weng AP , Ferrando AA , Lee W , et al. Activating mutations of NOTCH1 in human T cell acute lymphoblastic leukemia. Science. 2004;306(5694):269‐271. 15472075 10.1126/science.1102160

[15] O'Neil J , Grim J , Strack P , et al. FBW7 mutations in leukemic cells mediate NOTCH pathway activation and resistance to gamma‐secretase inhibitors. J Exp Med. 2007;204(8):1813‐1824. 17646409 10.1084/jem.20070876 PMC2118656

[16] Bandapalli OR , Schuessele S , Kunz JB , et al. The activating STAT5B N642H mutation is a common abnormality in pediatric T‐cell acute lymphoblastic leukemia and confers a higher risk of relapse. Haematologica. 2014;99(10):E188‐E192. 24972766 10.3324/haematol.2014.104992 PMC4181267

[17] Gianfelici V , Chiaretti S , Peragine N , et al. Prognostic impact of Jak/Stat, Ras/Akt and Notch1/Fbxw7 mutations in T‐cell acute lymphoblastic leukemia. Haematologica. 2015;100:20.

[18] Jenkinson S , Koo K , Mansour MR , et al. Impact of NOTCH1/FBXW7 mutations on outcome in pediatric T‐cell acute lymphoblastic leukemia patients treated on the MRC UKALL 2003 trial. Leukemia. 2013;27(1):41‐47. 22814294 10.1038/leu.2012.176

[19] Jenkinson S , Kirkwood AA , Goulden N , Vora A , Linch DC , Gale RE . Impact of PTEN abnormalities on outcome in pediatric patients with T‐cell acute lymphoblastic leukemia treated on the MRC UKALL2003 trial. Leukemia. 2016;30(1):39‐47. 26220040 10.1038/leu.2015.206 PMC4705426

[20] Pui CH , Yang JJ , Hunger SP , et al. Childhood acute lymphoblastic leukemia: progress through collaboration. J Clin Oncol. 2015;33(27):2938‐2948. 26304874 10.1200/JCO.2014.59.1636 PMC4567699

[21] Gokbuget N , Beck J , Brandt K , et al. Significant improvement of outcome In adolescents and young adults (AYAs) Aged 15–35 years with acute lymphoblastic leukemia (ALL) with a pediatric derived adult ALL protocol; results of 1529 AYAs in 2 consecutive trials of the German Multicenter Study Group For Adult ALL (GMALL). Blood. 2013;122(21):839.

[22] Ribera JM , Oriol A , Sanz MA , et al. Comparison of the results of the treatment of adolescents and young adults with standard‐risk acute lymphoblastic leukemia with the programa espanol de tratamiento en hematologia pediatric‐based protocol ALL‐96. J Clin Oncol. 2008;26(11):1843‐1849. 18398150 10.1200/JCO.2007.13.7265

[23] Rowe JM , Buck G , Burnett AK , et al. Induction therapy for adults with acute lymphoblastic leukemia: results of more than 1500 patients from the international ALL trial: MRC UKALL XII/ECOG E2993. Blood. 2005;106(12):3760–3767. 16105981 10.1182/blood-2005-04-1623

[24] Vidriales MB , Perez JJ , Lopez‐Berges MC , et al. Minimal residual disease in adolescent (older than 14 years) and adult acute lymphoblastic leukemias: early immunophenotypic evaluation has high clinical value. Blood. 2003;101(12):4695‐4700. 12586618 10.1182/blood-2002-08-2613

[25] Bruggemann M , Raff T , Flohr T , et al. Clinical significance of minimal residual disease quantification in adult patients with standard‐risk acute lymphoblastic leukemia. Blood. 2006;107(3):1116‐1123. 16195338 10.1182/blood-2005-07-2708

[26] Holowiecki J , Krawczyk‐Kulis M , Giebel S , et al. Status of minimal residual disease after induction predicts outcome in both standard and high‐risk Ph‐negative adult acute lymphoblastic leukaemia. The Polish Adult Leukemia Group ALL 4‐2002 MRD Study. Br J Haematol. 2008;142(2):227‐237. 18492099 10.1111/j.1365-2141.2008.07185.x

[27] Nikolova V , Shivarov V , Morilla R . Flow cytometric minimal residual disease levels after first inducton can define T‐acute lymphoblastic leukemia patients with high risk of relapse. Blood. 2012;120(21):4817.

[28] Beldjord K , Chevret S , Asnafi V , et al. Oncogenetics and minimal residual disease are independent outcome predictors in adult patients with acute lymphoblastic leukemia. Blood. 2014;123(24):3739‐3749. 24740809 10.1182/blood-2014-01-547695

[29] Trinquand A , Tanguy‐Schmidt A , Ben Abdelali R , et al. Toward a NOTCH1/FBXW7/RAS/PTEN‐based oncogenetic risk classification of adult T‐cell acute lymphoblastic leukemia: a group for research in adult acute lymphoblastic leukemia study. J Clin Oncol. 2013;31(34):4333‐4342. 24166518 10.1200/JCO.2012.48.5292

[30] Vega N , Malatesta R , Rives S , et al. Negative outcome in Pten mutated acute lymphoblastic leukemia pediatric patients could be modulated by the presence of Notch1/Fbxw7 mutations. Haematologica. 2014;99:10‐11.

[31] Ben Abdelali R , Buzyn A , Asnafi V , et al. NOTCH1/FBXW7 mutations, but not low ERG/BAALC expression, identify a major subgroup of adult T‐ALL with a favorable outcome: a GRAALL study. Blood. 2009;114(22):627‐628. 10.1182/blood-2011-02-334219 21835957

[32] Petit A , Trinquand A , Chevret S , et al. Oncogenetic risk classification based on NOTCH1/FBXW7/RAS/PTEN mutation profiles improves outcome prediction in pediatric T‐cell acute lymphoblastic leukemia, treated according the Fralle 2000 T guidelines. Blood. 2016;128(22):1083.

[33] Petit A , Trinquand A , Chevret S , et al. Oncogenetic mutations combined with MRD improve outcome prediction in pediatric T‐cell acute lymphoblastic leukemia. Blood. 2018;131(3):289‐300. 29051182 10.1182/blood-2017-04-778829

[34] Kadia TM , Gandhi V . Nelarabine in the treatment of pediatric and adult patients with T‐cell acute lymphoblastic leukemia and lymphoma. Expert Rev Hematol. 2017;10(1):1‐8. 10.1080/17474086.2017.1262757 PMC5578611 27869523

[35] Brown PA , Shah B , Fathi A , et al. NCCN guidelines insights: acute lymphoblastic leukemia, Version 1.2017. J Natl Compr Canc Netw. 2017;15(9):1091‐1102. 28874594 10.6004/jnccn.2017.0147

[36] Vega F , Medeiros LJ , Jones D , et al. A novel four‐color PCR assay to assess T‐cell receptor gamma gene rearrangements in lymphoproliferative lesions. Am J Clin Pathol. 2001;116(1):17‐24. 11447747 10.1309/5WFQ-N12E-DT05-UX1T

[37] Ok CY , Loghavi S , Sui D , et al. Persistent IDH1/2 mutations in remission can predict relapse in patients with acute myeloid leukemia. Haematologica. 2019;104(2):305‐311. 30171025 10.3324/haematol.2018.191148 PMC6355476

[38] Robinson JT , Thorvaldsdottir H , Winckler W , et al. Integrative genomics viewer. Nat Biotechnol. 2011;29(1):24‐26. 21221095 10.1038/nbt.1754 PMC3346182

[39] den Dunnen JT , Dalgleish R , Maglott DR , et al. HGVS recommendations for the description of sequence variants: 2016 update. Hum Mutat. 2016;37(6):564‐569. 26931183 10.1002/humu.22981

[40] Kox C , Zimmermann M , Stanulla M , et al. The favorable effect of activating NOTCH1 receptor mutations on long‐term outcome in T‐ALL patients treated on the ALL‐BFM 2000 protocol can be separated from FBXW7 loss of function. Leukemia. 2010;24(12):2005‐2013. 20944675 10.1038/leu.2010.203 PMC3035973

[41] Grossmann V , Haferlach C , Weissmann S , et al. The molecular profile of adult T‐cell acute lymphoblastic leukemia: mutations in RUNX1 and DNMT3A are associated with poor prognosis in T‐ALL. Genes Chromosomes Cancer. 2013;52(4):410‐422. 23341344 10.1002/gcc.22039

[42] Chiaretti S , Brugnoletti F , Tavolaro S , et al. TP53 mutations are frequent in adult acute lymphoblastic leukemia cases negative for recurrent fusion genes and correlate with poor response to induction therapy. Haematologica. 2013;98(5):e59‐e61. 23403321 10.3324/haematol.2012.076786 PMC3640132

[43] Salmoiraghi S , Montalvo ML , Ubiali G , et al. Mutations of TP53 gene in adult acute lymphoblastic leukemia at diagnosis do not affect the achievement of hematologic response but correlate with early relapse and very poor survival. Haematologica. 2016;101(6):e245‐e248. 26992948 10.3324/haematol.2015.137059 PMC5013945

[44] Kawamura M , Ohnishi H , Guo SX , et al. Alterations of the p53, p21, p16, p15 and RAS genes in childhood T‐cell acute lymphoblastic leukemia. Leukemia Res. 1999;23(2):115‐126. 10071127 10.1016/s0145-2126(98)00146-5

[45] Zuurbier L , Homminga I , Calvert V , et al. Meijerink, NOTCH1 and/or FBXW7 mutations predict for initial good prednisone response but not for improved outcome in pediatric T‐cell acute lymphoblastic leukemia patients treated on DCOG or COALL protocols. Leukemia. 2010;24(12):2014‐2022. 20861909 10.1038/leu.2010.204

[46] Clappier E , Collette S , Grardel N , et al. NOTCH1 and FBXW7 mutations have a favorable impact on early response to treatment, but not on outcome, in children with T‐cell acute lymphoblastic leukemia (T‐ALL) treated on EORTC trials 58881 and 58951. Leukemia. 2010;24(12):2023‐2031. 20861920 10.1038/leu.2010.205

[47] Zhu YM , Zhao WL , Fu JF , et al. NOTCH1 mutations in T‐cell acute lymphoblastic leukemia: prognostic significance and implication in multifactorial leukemogenesis. Clin Cancer Res. 2006;12(10):3043‐3049. 16707600 10.1158/1078-0432.CCR-05-2832

[48] Llorente LG , Luther H , Schneppenheim R , Zimmermann M , Felice M , Horstmann MA . Identification of novel NOTCH1 mutations: increasing our knowledge of the NOTCH signaling pathway. Pediatr Blood Cancer. 2014;61(5):788‐796. 24249312 10.1002/pbc.24852

[49] Marks DI , Paietta EM , Moorman AV , et al. T‐cell acute lymphoblastic leukemia in adults: clinical features, immunophenotype, cytogenetics, and outcome from the large randomized prospective trial (UKALL XII/ECOG 2993). Blood. 2009;114(25):5136‐5145. 19828704 10.1182/blood-2009-08-231217 PMC2792210

[50] Jain N , Lamb AV , O'Brien S , et al. Early T‐cell precursor acute lymphoblastic leukemia/lymphoma (ETP‐ALL/LBL) in adolescents and adults: a high‐risk subtype. Blood. 2016;127(15):1863‐1869. 26747249 10.1182/blood-2015-08-661702 PMC4915808

[51] Van Vlierberghe P , Ambesi‐Impiombato A , De Keersmaecker K , et al. Prognostic relevance of integrated genetic profiling in adult T‐cell acute lymphoblastic leukemia. Blood. 2013;122(1):74‐82. 23687089 10.1182/blood-2013-03-491092 PMC3701905

