# Supplementary figures and images for: RAS and TP53 can predict survival in adults with T‐cell lymphoblastic leukemia treated with hyper‐CVAD
Source: Cancer Med. 2019 Dec 5;9(3):849–58. doi: 10.1002/cam4.2757 (PMC6997098; doi:10.1002/cam4.2757)

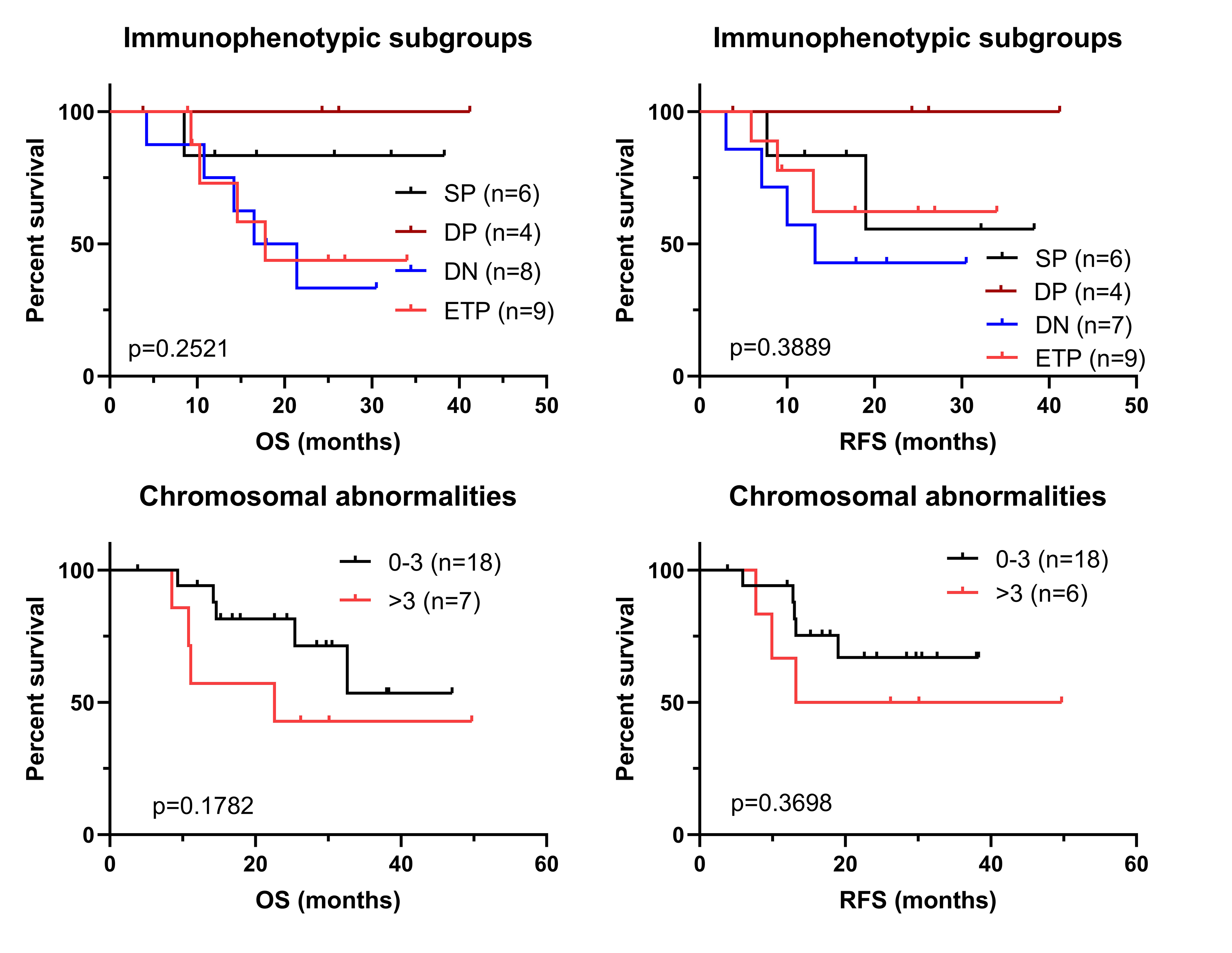

Supplement: Supplementary file 1 [file CAM4-9-849-s001.jpg]
