# Supplementary material for: RAS and TP53 can predict survival in adults with T‐cell lymphoblastic leukemia treated with hyper‐CVAD
Source: Cancer Med. 2019 Dec 5;9(3):849–58. doi: 10.1002/cam4.2757 (PMC6997098; doi:10.1002/cam4.2757)
Supplement: Supplementary file 2 [file CAM4-9-849-s002.docx]

Supplementary table - 1

| **P**  Pt. ID | T cell  phenotype | Chromosomal  Abnormality  (n) | CD1a | CD4 | CD8 | CD5 | CD13 | CD33 | CD117 | CD34 | HLA-DR |
| --- | --- | --- | --- | --- | --- | --- | --- | --- | --- | --- | --- |
| 1 | SP | 0 | pos | neg | pos | pos | neg | neg | neg | neg | neg |
| 2 | SP | na | pos | pos | neg | pos | neg | neg | neg | neg | neg |
| 3 | ETP | 2 | neg | neg | neg | partial | neg | neg | neg | pos | neg |
| 4 | DN | 1 | pos | neg | neg | pos | neg | neg | neg | pos | neg |
| 5 | DN | 5 | neg | neg | neg | pos | pos | neg | neg | pos | pos |
| 6 | DN | >5 | neg | neg | neg | pos | pos | neg | neg | pos | dim |
| 7 | DN | 4 | neg | neg | neg | pos | neg | pos | neg | pos | neg |
| 8 | SP | 0 | pos | pos | neg | pos | neg | neg | neg | pos | neg |
| 9 | DN | na | neg | neg | neg | pos | neg | neg | neg | neg | neg |
| 10 | SP | 0 | pos | pos | neg | pos | neg | neg | neg | neg | neg |
| 11 | DN | 2 | neg | neg | neg | pos | neg | pos | neg | pos | neg |
| 12 | DN | 0 | neg | neg | neg | pos | pos | neg | neg | pos | partial |
| 13 | DP | 5 | pos | pos | pos | pos | neg | neg | neg | neg | neg |
| 14 | DP | 5 | pos | pos | pos | pos | neg | neg | neg | neg | neg |
| 15 | ETP | 2 | neg | neg | neg | partial | neg | neg | pos | pos | neg |
| 16 | ETP | 0 | neg | pos | neg | neg | neg | neg | neg | neg | pos |
| 17 | DP | 0 | pos | pos | pos | pos | neg | neg | neg | neg | neg |
| 18 | DP | 0 | pos | pos | pos | pos | neg | neg | neg | neg | neg |
| 19 | ETP | 0 | neg | neg | neg | neg | pos | neg | pos | pos | pos |
| 20 | ETP | 0 | neg | neg | neg | neg | pos | neg | pos | neg | neg |
| 21 | DN | 3 | neg | neg | neg | pos | neg | pos | neg | pos | neg |
| 22 | ETP | 0 | neg | neg | neg | neg | pos | neg | pos | pos | pos |
| 23 | ETP | 3 | neg | neg | neg | neg | neg | pos | neg | pos | neg |
| 24 | ETP | >5 | neg | neg | neg | neg | pos | neg | pos | pos | pos |
| 25 | SP | 0 | neg | pos | neg | pos | neg | neg | neg | partial | neg |
| 26 | SP | 5 | neg | pos | neg | pos | neg | pos | neg | pos | pos |
| 27 | ETP | 0 | neg | neg | neg | neg | pos | neg | pos | pos | pos |

Immunophenotypic and cytogenetic characteristics of patient cohort. The immunophenotype is based on flow cytometry analysis of concurrent bone marrow aspirate. Chromosomal changes are based on conventional karyotyping.

DN: double negative for CD4 and CD8; DP: double positive for CD4 and CD8; ETP-ALL: early T-precursor acute lymphoblastic leukemia; NA: data not available; partial: level of expression is less than 25%; SP: single positive for CD4 or CD8.
